# Do Panic Symptoms Affect the Quality of Life and Add to the Disability in Patients with Bronchial Asthma?

**DOI:** 10.1155/2015/608351

**Published:** 2015-09-03

**Authors:** A. D. Faye, S. Gawande, R. Tadke, V. C. Kirpekar, S. H. Bhave, A. P. Pakhare, B. Tayade

**Affiliations:** ^1^Department of Psychiatry, NKP Salve Institute of Medical Sciences and Lata Mangeshkar Hospital, Digdoh Hills, Hingna Road, Nagpur, Maharashtra 440019, India; ^2^Department of Community and Family Medicine, All India Institute of Medical Sciences, Bhopal 462020, India; ^3^Department of Chest Medicine, NKP Salve Institute of Medical Sciences and Lata Mangeshkar Hospital, Digdoh Hills, Hingna Road, Nagpur, Maharashtra 440019, India

## Abstract

*Background*. Anxiety and panic are known to be associated with bronchial asthma with variety of impact on clinical presentation, treatment outcome, comorbidities, quality of life, and functional disability in patients with asthma. This study aims to explore the pattern of panic symptoms, prevalence and severity of panic disorder (PD), quality of life, and disability in them. *Methods*. Sixty consecutive patients of bronchial asthma were interviewed using semistructured proforma, Panic and Agoraphobia scale, WHO Quality of life (QOL) BREF scale, and WHO disability schedule II (WHODAS II). *Results*. Though 60% of the participants had panic symptoms, only 46.7% had diagnosable panic attacks according to DSM IV TR diagnostic criteria and 33.3% had PD. Most common symptoms were “sensations of shortness of breath or smothering,” “feeling of choking,” and “fear of dying” found in 83.3% of the participants. 73.3% of the participants had poor quality of life which was most impaired in physical and environmental domains. 55% of the participants had disability score more than a mean (18.1). *Conclusion*. One-third of the participants had panic disorder with significant effect on physical and environmental domains of quality of life. Patients with more severe PD and bronchial asthma had more disability.

## 1. Introduction

Asthma is a major public health problem and its prevalence is increasing in both developed and developing countries in recent years [1, 2]. As it is a chronic illness, it has psychological consequences also. Though studies have suggested an association between asthma (especially of severe grade) and mental disorders, there are very few Indian studies that demonstrate the association of asthma and panic disorder (PD). Few studies have found higher rates of anxiety disorders (particularly panic disorder) and major depression among adults with asthma [3–6]. Though there is an increased rate of psychological comorbidity in patients with asthma [7–9], anxiety can be a normal reaction to extreme breathing difficulty [10] and may even be functional in moderation [11, 12]. High levels of panic fear can lead to poor management. Panic and anxiety can directly exacerbate asthma symptoms through hyperventilation, patients' overuse of as-needed asthma medications, more frequent hospital admissions, and longer hospital stays and with more frequent steroid treatment. All of these are independent of degree of objective pulmonary impairment [13, 14].

It has been reported that the prevalence rate of PD is two to six times more among asthma patients compared to general population [15]. The reason for this observation is yet not clear. There is also a symptom overlap and many symptoms of acute asthma can be misunderstood for panic and vice versa. There is limited data on the impact of anxiety comorbidity on functional status and asthma quality of life [9]. Effects of asthma symptoms and physical limitations along with phobic avoidance of a feared situation or trigger factors all can lead to poor quality of life. Similarly there is a sociooccupational impairment in panic disorder. It is also shown that asthma patients with PD had greater perceived impairment from asthma and health care utilization [16]. The study of panic disorder among asthmatics can contribute to improved medical outcome for this subgroup, while also improving our understanding of the biological and psychological etiology of panic. This study was conducted to review this relationship by examining the pattern of panic symptoms, prevalence of panic disorder, how panic disorder and panic symptoms affect the course of asthma and quality of life (QOL) in these patients, and the consequent research implications of this relationship.

## 2. Material and Methods

A hospital based cross-sectional study was conducted in Psychiatry and Chest Medicine Outpatient Department of tertiary care hospital between January 2012 and June 2013. Considering logistic feasibility, we decided to enroll 60 consecutive asthma patients so that this study would act as pilot for further research. Patients were selected from outpatient department of chest medicine. Bronchial asthma was diagnosed and categorized according to the severity by a qualified chest physician in tertiary care hospital (where the study was carried out), on the basis of clinical history, pulmonary function test, and GINA (Global Initiative for Asthma) pocket guide updated in 2011. A chest physician identified asthma patients between 18 to 60 years of age who were maintained (stable) on treatment. Enrolment was consecutive. Then, these patients were informed about the study and referred to psychiatry outpatient department where a qualified psychiatrist ascertained eligibility criterion. Patients who had already been diagnosed with other axis I psychiatric disorders and those with active symptoms or having acute exacerbation of asthma and unable to give informed consent were excluded from the study.

The whole interview process was semistructured which included clinical assessment, clinical psychiatric assessment, semistructured pro forma questionnaire, and DSM IV TR (Diagnostic and Statistical Manual for diagnosing mental disorders, 4th edition revised version) criteria for diagnosis of panic attack and panic disorder. The panic attack symptoms were selected from the list of the symptoms for DSM IV TR criteria for panic attack. Then, those satisfying the DSM IV TR criteria for panic disorder were considered for the diagnosis of panic disorder. Panic disorder was assessed by clinical interview with patients and close family members by authors who are qualified psychiatrists and diagnosis is done with structured diagnostic criteria of DSM IV TR.

All patients were given detailed information of the study in local language and written informed consent was obtained before administering questionnaires.

Then following questionnaires were administered in order as described below. All the questionnaires used were in local language, that is, Marathi or Hindi. On an average, one interview lasted for 45 minutes. They were as follows:

(1)* Semistructured pro forma* includes sociodemographic data and details regarding asthma (duration, treatment, severity, comorbid medical illnesses, past and family history, history of substance use, and psychiatric history).

(2)* Diagnostic and Statistical Manual for diagnosing mental disorders, 4th edition revised version (DSM IV TR)* for assessment of panic symptoms and panic disorder [17]: panic attack is diagnosed if 4 or more of the symptoms, out of total 13 symptoms mentioned, are developed abruptly and reached a peak within 10 minutes, while panic disorder is diagnosed if there are recurrent unexpected panic attacks followed by one-month or more period of persistent concern about additional attacks or worry about the implications of the attack or significant change in behaviour related to the attack along with other criteria.

(3)* Panic and Agoraphobia scale (PAS)* [18] to assess the severity of panic disorder: it is a special instrument used to determine the severity of PD. The scale contains 13 questions (items) each with 5 possible answers (0–4). Five components have been taken into account: panic attacks, agoraphobic avoidance, anticipatory anxiety, disability, and worries about health. Severity is assessed as 0–8: lack of symptoms; 9–18: mild symptoms; 19–39: severe symptoms; 40 or more: very severe symptoms.

(4)* WHO Quality of Life BREF scale* [19] for assessment of quality of life: from the overall quality of life and General Health facet: domain scores are scaled in a positive direction (i.e., higher scores denote higher quality of life). The mean score of items within each domain is used to calculate the domain score. Mean scores are then multiplied by 4 in order to make domain scores comparable with the scores used in the WHOQOL-100. It is an abbreviated version of WHO-QOL 100 scale, containing 26 items each rated on a 5-point Likert scale. It measures quality of life in 4 domains: physical, psychological, social, and environmental along with two items. The raw scores are converted into transformed scores to obtain the domain scores in the range of 0–100. The scale has high internal consistency (Cronbach alpha = 0.94).

(5)* World Health Organization Disability Assessment Schedule II (WHODAS II)*: this is a multidimensional questionnaire for measuring the level of disability across various conditions and interventions. It explores different areas of functioning in daily life (e.g., hygiene, work, concentration, and relationships). The short version of the scale is a 12-item scale covering the following six domains:

1. Understanding and communicating with the world (cognition).
2. Moving and getting around (mobility).
3. Self-care (attending to one's hygiene, dressing, eating, and staying alone).
4. Getting along with people (interpersonal interactions).
5. Life activities (domestic responsibilities, leisure, and work).
6. Participation in society (joining in community activities).

Scoring is based on averaging responses and then transforming scores into a standard scale. Two types of scoring (ways to compute the summary scores), namely, simple and complex scoring, are available. In simple scoring, the scores assigned to each of the items (none, 1; mild, 2; moderate, 3; severe, 4; and extreme, 5) are summed up without recoding or collapsing response categories. In complex scoring, also known as item response theory-based scoring, multiple levels of difficulty for each WHODAS 2.0 item are allowed for. Each item response (none, mild, moderate, severe, and extreme) is treated separately and the summary score is generated with a computer by differentially weighting the items and the levels of severity. Overall test-retest reliability of the scale is 0.94; reliability of each of the six domains ranges from 0.86 to 0.92.

## 3. Data Analysis

Data were entered in MS-Excel and analyzed in IBM SPSS version 21 (IBM Inc.) software. Categorical variables were summarized as counts and proportions, while numerical variables were summarized as mean and standard deviation. Prevalence was reported as proportion with its 95% confidence interval. Sociodemographic details, clinical data and scores of various scales were compared between asthmatics with and without panic disorder using Chi-square and unpaired *t*-test appropriately. Scores across the various domains of scales where weightage for each domain was equal were compared by related measure ANOVA which is popularly known as repeated measure ANOVA. A *p* value less than 0.05 was considered statistically significant.

## 4. Results

A total of 73 patients of asthma were screened for eligibility and administered consent to achieve desired sample size of 60 willing participants.  Table 1 demonstrates the demographic profile of the study participants (Table 1).

Table 2 shows the profile of asthma with respect to duration, treatment taken, family history, and any substance abuse. Mean duration of bronchial asthma in this study group was 7.8 years. 66.7% (40 out of 60) of the participants were on treatment with both inhaler and oral drugs whereas 33.3% (20) were on inhaler only. 26.7% (16) of the patients were on (oral) steroid treatment. The majority (66.7%, i.e., 40) of the participants had history of multiple hospitalizations for asthma. Family history of bronchial asthma was found in only (12) 20% of the subjects and only 4 participants (6.7%) had history of psychiatric illness in the family. History of smoking was found in 53.3% (32) of the subjects while only 8 subjects (13.3%) had history of alcohol consumption. Other comorbid medical conditions like hypertension and chronic obstructive pulmonary disease were present in only 6.7% (4) of the subjects. Sixty percent (36) of the participants were suffering from moderate grade asthma whereas 33.3% (20) had mild asthma and only 4 (6.7%) had severe asthma (Table 2).

This study (Table 3) that 33.3% (20) (95% CI 22.7 to 45.9) of the participants had panic disorder (PD), 16.7% (10) (95% CI 9.3 to 28.0) had adjustment disorder, and 13.3% (8) (95% CI 6.9 to 24.1) suffered panic attacks only (not fulfilling the criteria for panic disorder). Around 20% (12) (95% CI 11.8 to 31.8) did not have any diagnosable psychiatric disorder. Others had mild depression and mixed anxiety depression (Table 3).

60% (36) of the patients had 4 or more panic symptoms (not satisfying the criteria for panic attack). Out of this group, the most common symptoms were “sensations of shortness of breath or smothering,” “feeling of choking,” and “fear of dying” found in 83.3%. As these symptoms are similar to those of acute attack of asthma, these patients thought they have an acute episode of asthma while experiencing the above symptoms but they simultaneously explained some other symptoms which made us diagnose them as panic symptoms. Other symptoms were chest discomfort (72.2%), palpitations (58.33%), sweating (50%), tingling sensations in the extremities (47.2%), trembling of body (41.67%), feeling dizzy and unsteady (36.11%), and feeling of losing control (30.55%).

58.3% (28) of the patients with psychiatric diagnosis required treatment with psychotropic medications (Selective Serotonin Reuptake Inhibitors (SSRIs) and short course of Benzodiazepines (BZDs)) whereas 41.7% (20) were started on psychotherapy and BZDs on “as and when needed” basis. Though symptoms pattern was variable, 28 (46.7%) patients (out of total 60 patients) had diagnosable panic attack (only panic attacks (13.3%) + panic disorder (33.3%)) according to DSM IV TR. Out of this group, 57.1% (16 out of 28) had more than 4 panic attacks during the course of asthma. When patients of panic disorder were assessed for severity with Panic and Agoraphobia scale (PAS), 70% (14 out of 20) were found having “severe PD” whereas 25% (5 out of 20) had “mild PD.”

In this study, 4 participants had panic symptoms (not diagnosable with panic attacks or panic disorder) before they developed asthma, out of which 3 had aggravation of panic symptoms during/after every attack of acute asthma. Subsequently, one turned up into panic disorder and the other two were diagnosed as having panic attacks. Out of 4, one continued to show panic symptoms only (not diagnosable with panic attacks or panic disorder). Further, it could not be differentiated whether panic symptoms newly developed in asthma patients were due to reaction to the severity of asthma or induced by asthma medication.

## 5. Quality of Life

In this study, 44 (73.3%) of the participants had poor quality of life with the total score of less than 200 on WHOQOL BREF scale (mean: 195.5 and standard deviation: 20.4). The mean score of disability on WHODAS II was 18.1 (standard deviation: 9.2). 55% (33) of the subjects had disability score of more than 18.1 (Table 4).

We also compared the scores of each domain of QOL among the participants with and without panic disorder. This was done by related measure ANOVA popularly known as repeated measure ANOVA. QOL scores among those without panic disorders were similar across all the domains. However, QOL scores were statistically significantly lower in physical and environmental dimensions among those with panic disorder (Table 5).

Though statistically nonsignificant, the majority of patients with moderate and severe asthma had quality of life score of less than 200. All the subjects with severe panic disorder as per the PAS scores were having poor quality of life with score of less than 200 which was statistically significant. This study also showed that the mere presence of asthma and absence of panic disorder can also lead to poor quality of life as indicated by 63.6% of the participants without panic disorder having QOL score less than 200 (Table 6).

On comparing the quality of life with disability (though statistically not significant), it was found that the more the disability score, the poorer the quality of life (score < 200) (Table 7).

Statistically significant difference was found between WHODAS score and severity of panic disorder and asthma. The more severe the panic disorder or asthma, the higher the disability (*p* < 0.01).

## 6. Discussion

Asthma is a major health problem, with increasing prevalence, morbidity, and mortality in the last few decades. It is also among the 4 most common chronic disorders in adult population, with a prevalence of approximately 5% [20]. Along with the environmental factors like indoor allergens, psychological factors are increasingly recognized to influence the onset and course of asthma. Literature shows that the prevalence of panic disorder is much more in patients with asthma compared to general population. This study is among the very few Indian studies to focus on this association with study of symptom profile and the prevalence of panic disorder in asthma and its effects on quality of life and functional disability.

The mean age of participants in this study was 37 ± 7.4. In a study [21] titled Demographic and Environmental Factors in Patients of Bronchial Asthma, including 398 subjects, mean age of the subjects was found as 35.5 years ± 12.19 years with the range between 18 and 60 years. Most of the participants were married and this was explainable by the mean age of the study population. 46.7% of the subjects were doing unskilled work which may be due to the rural and suburban population in the catering area of the institute. Family history of bronchial asthma was found in only 12% of study population. This finding was contradictory to the findings of other studies. Some studies documented that at least one of the first-degree family members may have asthma, while in few other studies family history of bronchial asthma was observed in 82.1% of patients [22]. We found low prevalence of family history of psychiatric illness in this study. In this study sample, the data related to family history was collected based on the information given by the patients, wherein likelihood of underreporting or not knowing on the part of the participants resulting in lesser prevalence of family history of psychiatric illness is possible.

Mean duration of bronchial asthma in this study was 7.8 ± 4 which suggests the chronic nature of the illness. The majority of the participants were on combination of inhaler and oral medications as 60% of the subjects had moderate grade asthma which is difficult to be managed by inhaler only. Also most of the subjects had history of multiple hospitalizations.

More than half of the subjects had a history of smoking exposure (active or passive). In this study, we found that one-third of the participants had diagnosable panic disorder; another common psychiatric diagnosis was adjustment disorder. Previous data from the studies in clinical and community settings suggest that asthma and mental disorders cooccur more often than expected by chance [23–29]. Several studies have found increased prevalence of anxiety disorder, including panic attacks and symptoms of generalized anxiety disorder (GAD), among clinical samples of patients with asthma [28, 30].

The prevalence of panic disorder in adults is 1% to 3% in community population [31] and 4% to 8% in primary care populations [32]. Many cross-sectional studies in adult population have suggested that the prevalence of panic disorder among patients with asthma ranges from 6.5% to 24% [9, 29].

Most of the adult and child studies of patients with asthma have shown an increased rate of comorbid anxiety disorders [28]. Studies have also shown a relatively specific association between the prevalence of bronchial asthma and panic [33, 34]. The association is specific even after adjusting the differences in demographics and psychiatric comorbidity. Severe asthma is associated with even greater likelihood of panic attacks [33]. Asthma medications can also have anxiogenic properties [35], and anxiety may further enhance the use of asthma medication.

We also found that, among the subjects with panic disorder, around 70% had severe PD which implicates the need to assess and treat the panic disorder, if associated with bronchial asthma.

A large community sample of 4181 adults from Germany, which included physician confirmation of asthma using a physician interview, found that current severe asthma (in the last 4 weeks) was associated with a significantly increased likelihood of having any anxiety disorder including panic disorder. Lifetime severe asthma was also associated with an increased risk of having any anxiety disorder, panic disorder, social phobia, generalized anxiety disorder, and bipolar disorder [13]. Anxiety and depressive disorders are even more common in patients who present with severe life-threatening episodes of asthma as well as those hospitalized with acute asthma exacerbations [36].

In this study, mixed anxiety depression and mild depression were found in around 16.7% of the participants.

Sixty percent of the subjects had some or other panic symptoms which suggests that asthma and panic have strong correlation. Literature shows that this relation can be direct (as the asthmatic attack may mimic panic attack, medications used to treat asthma may cause panic symptoms, and chronicity of the illness also contributes to it) or indirect as panic can be a psychological reaction to the asthma episode and fear about future asthmatic attack.

In this study, we found that the quality of life was impaired (poor) in the majority of the subjects specifically in physical and environmental dimensions. It was also found that presence of panic disorder was associated with significantly lower quality of life in physical and environmental domains. Though the correlation between QOL total score and presence of PD was not significant, the severity of panic disorder was significantly correlated with poor (overall) quality of life. The (feared situation) avoidant behaviour along with the physical limitation due to asthma symptoms may be severely disabling and isolating. This may lead to the functional restriction much greater than objectively measured physiologic level of impairment [37]. It may be speculated that anxiety, panic, and fear avoidance can further manifest with depressive symptoms leading to further deterioration in quality of life. Depressive symptoms are known to affect treatment adherence and lead to a vicious cycle of poorer asthma control and quality of life [38]. This in turn may be manifested in increased utilization of health resources, as suggested by a study including 1406 adult asthma patients in whom the asthma quality of life significantly predicted subsequent Emergency Department utilization [39]. Thus, poor asthma quality of life has an impact on treatment and asthma control along with many aspects of the individual's personal and social life. In turn, it can also be said that better treatment of asthma may also be worth achieving, if it is the asthma that drives the panic.

The mean score on disability scale was 18.1 and more than half of the participants had scored more than 18.1. Panic disorder severity was also associated with more disability. The disability score was still higher in those with impaired quality of life (score ≤ 200). Quality of life in turn was impaired more with moderate to severe asthma and associated severe panic disorder. These correlation findings can be explained, as asthma and panic disorder and its severity can cause physical disability and limitations in social and routine activities (because of the symptom profile and effect of capacity of a person) and overall functioning which in turn affects the quality of life. Literature shows that the negative impact of asthma is usually estimated on the basis of mortality, number of asthma attacks, and number of hospitalizations. However, the effects of the asthma can impair other important aspects, such as the quality of life and physical well-being of patients and can affect performance at school or work [40, 41].

## 7. Conclusion

Most common panic symptoms found in this study population were “sensations of shortness of breath or smothering,” “feeling of choking,” and “fear of dying” (found in 83.3% of those with panic symptoms). 46.7% of the participants had diagnosable panic attack according to DSM IV TR and more than half of them had more than 4 panic attacks during the course of asthma.

Panic disorder was found in about 1/3rd of the participants out of which the majority had severe panic disorder. Quality of life was poor in the majority of the subjects with statistically significant impairment in physical and environmental domains found in those with severe PD. Patients with more severe panic disorder and bronchial asthma had more disability. Poor quality of life was found to have positive correlation with disability.

This is an important study to document the extent of comorbidity and whether comorbid anxiety, especially panic disorder, is associated with higher symptom load, impaired functioning, and poor quality of life.

As the comorbid rate was high, future studies need to be planned to enhance the understanding of biological and cognitive mechanisms that might explain this association. Large-scale studies are needed for patients with asthma and panic disorder to help validate the cooccurrence of these 2 conditions by measuring the effect of comorbidity on symptom burden, functioning, adherence to asthma self-care regimens, quality of life, and medical costs for controlling asthma.

## Figures and Tables

**Table 1:** Demographic profile of the participants.

Demographic factors		
Age in years	[Mean (SD)]	37 ± 7.4

Gender	Females	40% (24)
	Males	60% (36)

Marital status	Married	86.7% (52)
	Unmarried	13.3% (8)

Education status	Nil	10% (6)
	Up to primary	20% (12)
	Up to matriculation	43.3% (26)
	More than matriculation	26.7% (16)

Occupation status	Housewives	33.3% (20)
	Semiskilled	13.3% (8)
	Skilled	6.7% (4)
	Unskilled	46.7% (28)

**Table 2:** Profile of asthma patients.

Variable	Number	Percent
Duration of asthma [mean (SD), years]	7.8	(4.0)
Treatment received		
Oral drugs and inhaler	40	(66.7)
Only inhaler	20	(33.3)
On steroid treatment	16	(26.7)
≤3 years	4	(25.0)
4–6 years	3	(18.8)
7–9 years	5	(31.3)
10–12 years	4	(25.0)
Hospitalizations		
Nil	4	(6.7)
1 to 5	16	(26.7)
Multiple (>5)	40	(66.7)
Family H/O asthma	12	(20.0)
Family H/O psychiatric disorder	4	(6.7)
H/O of exposure to smoking	32	(53.3)
H/O of alcohol consumption	8	(13.3)
Presence of medical comorbidity	4	(6.7)
Severity of asthma		
Mild	20	(33.3)
Moderate	36	(60.0)
Severe	4	(6.7)

**Table 3:** Profile of psychiatric problems and panic symptoms.

Variable	Number	Percent
Psychiatric problems (*n* = 60)		
Nil	12	(20.0)
Adjustment disorder	10	(16.7)
Mixed anxiety depression	4	(6.7)
Mild depression	6	(10.0)
Panic attacks (only)	8	(13.3)
Panic disorder	20	(33.3)
H/O of psychiatric treatment (*n* = 48)		
Benzodiazepines (BZDs), psychotherapy	20	(41.7)
Selective Serotonin Reuptake Inhibitors, BZDs	28	(58.3)
Presence of any panic symptom (*n* = 60)	36	(60.0)
Number of panic symptoms (*n* = 36)		
4	2	5.6
5	5	13.9
6	9	25.0
7	11	30.6
8	7	19.4
9	2	5.6
Number of panic attacks (*n* = 28)		
2	4	14.3
4	8	28.6
5 or more	16	57.1
Severity of panic disorder (*n* = 20)		
Mild	5	25.0
Severe	14	70.0
Very severe	1	5.0

**Table 4:** WHO QOL BREF scale and WHODAS II mean scores.

QOL domains	Mean	SD
QOL total score	195.5	(20.4)
QOL physical	47.6	(5.3)
QOL psychological	50.5	(7.9)
QOL social	51.1	(6.5)
QOL environmental	46.2	(4.7)
QOL-total		
<200—73.3% (44)		
>200—26.75% (16)		
WHODAS II	18.1	(9.2)
≤18.1—45% (27)		
>18.1—55% (33)		

**Table 5:** Association between panic disorder and WHO Quality of Life BREF scale domains.

QOL dimensions	Mean	95% confidence interval	
		Lower	Upper
No panic disorder			
Physical	49.3	47.8	50.8
Psychological	51.8	49.3	54.2
Social	50.7	48.6	52.7
Environmental	47.6	46.3	49.0
Presence of panic disorder			
Physical	**44.3**	42.1	46.4
Psychological	47.9	44.4	51.4
Social	52.1	49.2	55.0
Environmental	**43.5**	41.5	45.4

**Table 6:** Association between severity of asthma and PD with QOL.

	QOL				*p* value
	<200		>200		
	Number	Percent	Number	Percent	
Severity of asthma					
Mild	12	27.3%	8	50.0%	
Moderate	28	63.6%	8	50.0%	
Severe	4	9.1%	0	0.0%	0.162
Presence of PD					
No	28	63.6%	12	75.0%	
Yes	16	36.4%	4	25.0%	0.409
PAS					
Mild	1	2.3%	4	25.0%	
NA	28	63.6%	12	75.0%	
Severe	14	31.8%	0	0.0%	**0.005 **
Very severe	1	2.3%	0	0.0%	

**Table 7:** Comparison between WHODAS II and QOL.

	QOL					
	<200		>200		Total	
	Mean	SD	Mean	SD	Mean	SD
WHODAS	20.91	8.32	10.31	6.98	18.08	9.23

## Limitations

Though this study is among the very few Indian studies to focus on symptom profile and prevalence of panic disorder in asthma, there are few limitations of the study that cannot be overlooked. The sample size of this study was small for the aim of finding the prevalence of panic disorder in patients with asthma. Secondly as the study was carried out in only one centre with a nonrandom selection of sample, it is hard to determine what a true point prevalence of panic disorder is. As this was a cross-sectional single interview study, the answers to the questionnaire might have been affected by the state of mind at the time of interview and no follow-up assessment was done. Psychiatric comorbidities that may be present along with the panic disorder were not analyzed statistically. Lastly, though it was expected that patients attending a tertiary referral centre are likely to be on medium to higher grade of treatments, details about the asthma medications, exact doses, and compliance could not be analyzed statistically for the correlation.
